# Fertility treatment and breast-cancer incidence: meta-analysis

**DOI:** 10.1093/bjsopen/zrab149

**Published:** 2022-02-09

**Authors:** Carolyn Cullinane, Hannah Gillan, James Geraghty, Denis Evoy, Jane Rothwell, Damian McCartan, Enda W. McDermott, Ruth S. Prichard

**Affiliations:** 1 Department of Breast Surgery, St Vincent’s University Hospital, Dublin, Ireland; 2 Department of Surgical Research, University of College Cork, Cork T12 K8AF, Ireland

## Abstract

**Background:**

The significance of exogenous hormone manipulation as part of fertility treatment and its relationship to the development of breast cancer remains uncertain. Several historical reviews have been performed with conflicting results. This study is an updated meta-analysis to determine whether there is a causal relationship between different fertility treatments and breast cancer.

**Methods:**

The study report is based on the guidelines of PRISMA and Meta-Analysis of Observational Studies in Epidemiology. Studies published within the last 20 years were included to reflect up to date *in vitro* fertilization (IVF) practice. This study was prospectively registered on PROSPERO on 07/04/2021, registration identification CRD42021247706. The primary outcome of the study was to determine whether there is an increased incidence of breast cancer in women treated with hormonal fertility treatment. The secondary outcomes were to determine whether fertility treatments were individually associated with excess breast-cancer risk.

**Results:**

Overall, 25 studies, including 617 479 participants, were eligible for inclusion. There was no significant breast-cancer risk association with fertility treatment (compared with general and subfertility reference groups). Summary odds ratio of all included studies was 0.97 (95 per cent c.i. 0.90 to 1.04). Women who received six or more IVF cycles did not have an increased risk of breast cancer. Similarly, there was no excess breast-cancer risk associated with clomiphene, human chorionic gonadotropin, gonadotropin analogues and progesterone when examined individually. Comparably, there was no significant association between fertility treatment and excess breast-cancer risk in patients with more than 10 years’ follow-up. Summary odds ratio was 0.97 (95 per cent c.i. 0.85 to 1.12).

**Conclusion:**

This meta-analysis did not find a significant association between fertility treatments and excess breast-cancer risk. Women considering IVF should be informed that it does not appear to increase breast-cancer risk.

## Teaser

This meta-analysis of 25 studies published within the last 20 years did not find a significant association between fertility treatments and excess breast-cancer risk. Women considering IVF should be informed that IVF does not appear to increase breast-cancer risk.

## Introduction

Breast cancer is the most common malignancy amongst reproductive women worldwide^1^. Prolonged oestrogen exposure is an established risk factor for developing oestrogen-positive breast cancer^2^. Since the first *in vitro* fertilization (IVF) baby was born in 1978, the use of IVF has increased exponentially^3^. It is estimated that at least 9 per cent of couples experience some form of infertility and 56 per cent of these couples will seek medical treatment for this^4^. In 2010, 1 per cent of all children born in the USA, 2 per cent of children in the UK and almost 4 per cent in Denmark and Finland were conceived through IVF^5^. The steady increase in IVF use can be attributed to the significant postponement of child-bearing in the western world^6^. The mean maternal age at first birth is trending upwards with the mean age approaching 30 years in several European countries and many women are delivering their first child aged 35 years or older^7^. As nulliparity and delayed childbearing are risk factors for developing breast cancer due to excess endogenous oestrogen exposure, there is significant anxiety surrounding the potential confounding risk of IVF^8,9^.

The significance of exogenous hormone manipulation for fertility treatment is a conflicting area. In contrast to hormone replacement therapy (HRT), fertility treatment induces transient high levels of circulating oestrogen to initiate ovulation in women with anovulatory disorders and control hyperstimulation in women receiving assisted reproductive technology (ART)^10^. Such therapies include ovarian-stimulating agents such as clomiphene, human chorionic gonadotropin (HcG) and gonadotropin analogues. They are administered during the follicular phase of the menstrual cycle to increase the serum concentration of gonadotropins, targeting follicle maturation and ovulation^11^. Ovulation induction is just one aspect of fertility treatment. To prevent spontaneous ovulation and hyperstimulation, continuous gonadotropin-releasing hormone (GnRH) analogue therapy is sometimes administered to suppress pituitary gonadotropin release. Progesterone therapy is also supplemented to support the luteal phase of ovulation in the presence of GnRH analogues^12,13^. Several reviews have been performed to determine whether there is a causal association between breast cancer and fertility treatment. In 2013, Sergentanis and colleagues did not find a correlation between overall breast-cancer risk and IVF, however they reported that women who received IVF aged less than 30 years had marginally adverse outcomes (pooled effect estimate 1.64 (95 per cent c.i. 0.96 to 2.80))^14^. In the same year, another meta-analysis conducted by Li and co-workers reported a protective effect of fertility treatment in the subgroup of breast-cancer patients (risk ratio 0.79 (95 per cent c.i. 0.65 to 0.95))^15^. A more recent review by Gennari and co-workers in 2015 reported an increased risk of breast cancer with longer follow-up (less than 10 *versus* greater than 10 years) in patients who had fertility treatment^10^.

Since publication of these reviews, several large population-based studies have been conducted. Van den Belt-Dusebout and colleagues studied a population of 25 108 women and reported that breast-cancer risk amongst IVF-treated women was not significantly different from that of women with subfertility who did not receive IVF, with a median follow-up of 21 years^16^. Similarly, a recent study conducted in the UK of over 250 000 women did not report any excess overall breast-cancer risk with IVF but suggested an increased incidence of breast carcinoma *in situ* with increasing number of cycles^17^. In contrast Reigstad and colleagues studied a cohort of women from the Medical Birth Register in Norway and found that women exposed to ART had an elevated risk of breast cancer, which became more apparent with greater than 10 years’ follow-up^18^. As a result of the current conflicting literature, an updated meta-analysis has been performed to determine whether there is a causal association between different fertility therapies and breast cancer.

## Methods

**Fig. 1:**
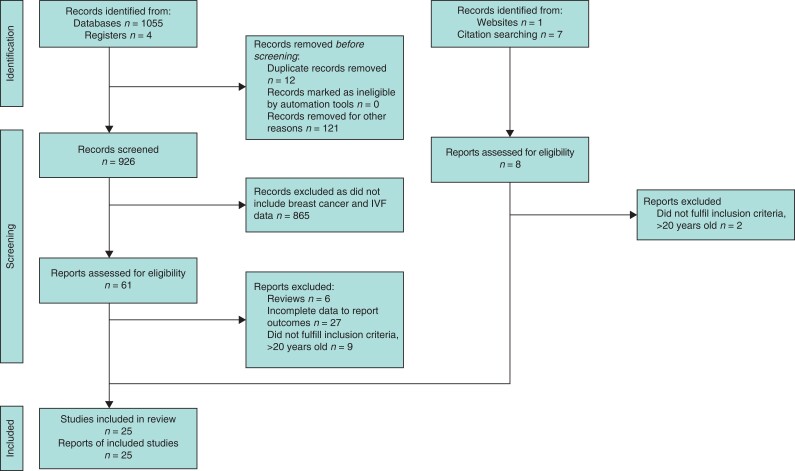
PRISMA flow diagram

The study report is based on the guidelines of PRISMA and Meta-Analysis of Observational Studies in Epidemiology^19^ (*Fig. 1*). Analysis and results were extracted from previous ethically approved studies therefore patient consent and ethical approval were not required. This study was registered prospectively on PROSPERO on 7 April 2021, registration identification CRD42021247706.

### Search strategy

An electronic search was conducted using the Cochrane library, Sciencedirect, PubMed and Embase. All studies from January 2000 to January 2021 were included. The following search terms/MESH terms were used: (IVF (Mesh) OR *in-vitro* fertilisation OR *in vitro* fertilisation OR fertility treatment OR assisted reproductive technology OR clomiphene) AND (Breastcancer (Mesh) OR breast cancer OR breast tumour OR breast carcinoma) AND (Incidence (Mesh) OR risk). All titles were initially screened, and appropriate abstracts were reviewed. Each of the publications’ bibliographies and Google Scholar were manually searched for relevant articles. The last date of search was 31 March 2021.

### Outcomes

The primary outcome was to determine whether there is an increased incidence of breast cancer in women treated with hormonal fertility treatment. The secondary outcomes were to determine whether individual fertility treatments such as clomiphene, HcG, gonadotropins and progesterone were associated with excess breast-cancer risk. Subgroup analysis was performed to elucidate whether increasing number of IVF cycles (six or more cycles) and longer duration of follow-up were associated with an increased incidence of breast cancer. Six cycles or more was the arbitrary cut-off point due to previous data suggesting an association between breast carcinoma *in situ* and more than five cycles of IVF^17^. Subgroup analysis of studies that used subfertility cohorts as reference groups was also performed to reduce potential confounding. The quality of the studies included was assessed using the Newcastle–Ottawa scale^20^. The risk of bias was assessed using the Risk of Bias in Non-Randomized Studies of Intervention Tool^21^. Publication bias was assessed using visual inspection of the forest plots.

### Inclusion criteria

To be included in the analysis, the studies had to meet the following criteria: subjects received fertility treatment and breast-cancer incidence expressed and publication date within the last 20 years to reflect more up-to-date IVF practice.

### Exclusion criteria

Studies were excluded from the analysis if published in languages other than English (for English proficiency reasons), if patients with genetic mutations increasing individuals’ risk of developing breast cancer (such as *BRCA* mutation carrier) were included, and if they did not present standardized incidence ratio, odds ratios, risk ratios or hazard ratios estimates (with 95 per cent confidence intervals, standard errors), or number of events necessary to calculate these for the outcomes of interest. Studies that included patients with a previous personal history of breast cancer, and those that included non-hormonal ART only (artificial insemination, surrogacy) were also excluded.

### Data extraction

Two reviewers (C.C., H.G.) independently reviewed the available literature according to the above predefined strategy and criteria. Each reviewer extracted the following variables: title and study details (year, design, country), study population characteristics (sample size, fertility treatment, number of cycles, follow-up, reference group, ART implantation method).

### Statistical analysis

Statistical analysis was performed using Review Manager 5 (The Cochrane Collaboration, The Nordic Cochrane Centre, Copenhagen, Denmark). Binary outcome data were reported as risk ratios with 95 per cent confidence intervals using the Mantel–Haenszel method. Risk ratios or odds ratios reported in the study publication were used when available; otherwise, they were extrapolated from the available data. Weighted mean differences were calculated for the effect size on continuous variables. Odds ratio greater than 1.00 indicated higher breast-cancer risk with fertility treatment.

Heterogeneity was assessed by *I*^2^ statistics, with greater than 50 per cent being considered significant heterogeneity. A fixed-effects model was preferred to a random-effects model when there was no significant heterogeneity and *vice versa* when there was significant heterogeneity (*I*^2^ greater than 50 per cent). Pooled estimates of differences were calculated using random-effects models, accounting for potential interstudy heterogeneity. *P* <0.050 was considered significant.

## Results

### Characteristics of the included studies

Overall, 25 studies, including 617 479 participants, were eligible for inclusion for analysis^12,16–18,22–42^ (*Fig. 1*, *Table S1*). Two studies were prospective cohort studies^28,39^ and two studies were case–control studies^23,38^. The other 21 studies were historical cohort studies^12,16–18,22,24–27,29–37,40–42^. Studies from Asia, Europe, Australia and North America were included. Twenty-one studies reported the incidence of breast cancer amongst women who received fertility treatment^12,16–18,22,24–26,28–37,40–42^, whilst the other four studies examined the number of patients with breast cancer who received fertility treatment using a reverse causation approach^23,27,38,39^. Study follow-up periods ranged from 3.6–30 years with seven studies including data with over 10 years of follow-up^16,18,24,29,31,36,40^. All 25 studies included multiple regimens of IVF^12,16–18,22–42^. Ten studies used women with infertility as a reference group^12,16,17,22,29–31,36,37,41^, 12 studies used the general population as a comparison^18,24–26,28,32–35,39,40,42^ and the remaining three studies examined a cohort of women with breast cancer who did or did not have a history of IVF use^23,27,38^.

### Overall breast-cancer risk associated with all fertility treatment

**Fig. 2:**
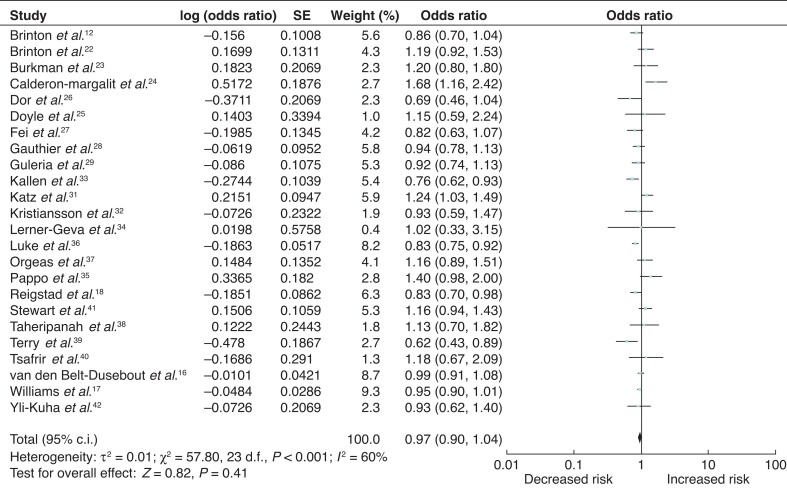
Forest plot of breast-cancer risk with all fertility treatments

Twenty-four studies were eligible for inclusion for analysis of overall breast-cancer risk associated with all fertility treatment^12,16–18,22–29,31–42^. Studies including the general population and infertile cohorts as reference groups were pooled together. Date of publication ranged from 2001 to 2020. The odds ratios for each of these studies and the summary odds ratio are shown in *Fig. 2*. There was no significant breast-cancer risk association with fertility treatment. Summary odds ratio was 0.97 (95 per cent c.i. 0.90 to 1.04). Heterogeneity testing demonstrated significant heterogeneity between the studies (*I*^2 ^= 60 per cent; *P *< 0.001) (*Fig. 2*).

### Breast-cancer risk associated with clomiphene fertility treatment

**Fig. 3:**
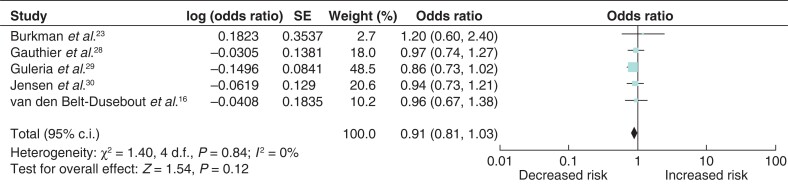
Forest plot of breast-cancer risk associated with clomiphene

Nine studies reported the incidence of breast cancer in women who received the ovulation induction agent clomiphene^12,22–24,28–30,37,38^. The odds ratios for each of these studies and the summary odds ratio are shown in *Fig. 2*. There was no significant association between breast cancer risk and clomiphene administration, odds ratio 1.07 (95 per cent c.i. 0.98 to 1.16). There was no significant heterogeneity between the studies (*I*^2^ = 15 per cent; *P* = 0.31) (*Fig. 3*).

### Breast-cancer risk associated with human chorionic gonadotropin fertility treatment

**Fig. 4:**
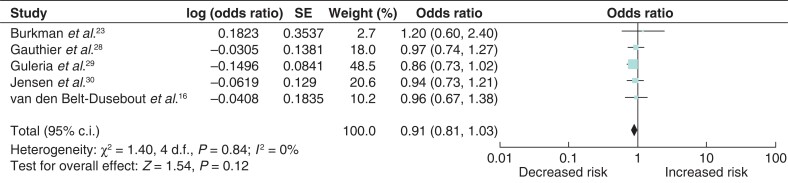
Forest plot of breast-cancer risk associated with human chorionic gonadotropin

Five studies were eligible for inclusion in the analysis of breast-cancer risk associated with HcG fertility treatment^16,23,28–30^. Similarly, there was no significant association between breast-cancer risk and HcG fertility treatment (odds ratio 0.91 (95 per cent c.i. 0.81 to 1.03)) with no heterogeneity between the studies (*Fig. 4*). However, one study constituted 48.5 per cent of the weighted mean.

### Breast-cancer risk associated with progesterone fertility treatment

**Fig. 5:**
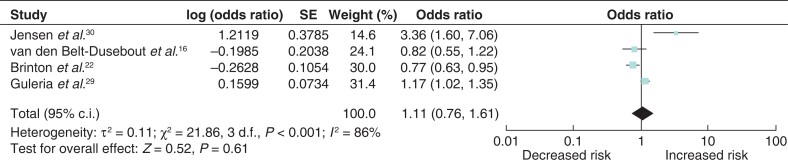
Forest plot of breast-cancer risk associated with progesterone

Four studies examined the association between breast-cancer incidence and progesterone fertility treatment^12,16,29,30^. Breast-cancer risk was not significantly associated with progesterone treatment (odds ratio 1.11 (95 per cent c.i. 0.76 to 1.61)). Significant heterogeneity was observed between the studies (*I*^2^ = 86 per cent, *P* < 0.001) (*Fig. 5*).

### Breast-cancer risk associated with gonadotropin fertility treatment

**Fig. 6:**
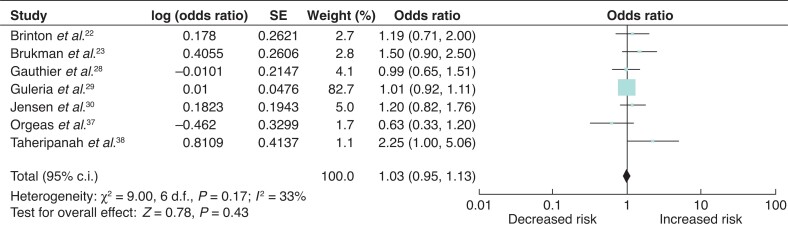
Forest plot of breast-cancer risk associated with gonadotropins

The associated risk between breast-cancer incidence and gonadotropin fertility treatment was examined in seven studies, with one study contributing 82.7 per cent to the overall weight mean^22,23,28–30,37,38^. There was no significant association between gonadotropin analogues and breast-cancer incidence (odds ratio 1.03 (95 per cent c.i. 0.95 to 1.13)) (*Fig. 6*).

### Breast-cancer risk associated with six or more cycles of IVF

**Fig. 7:**
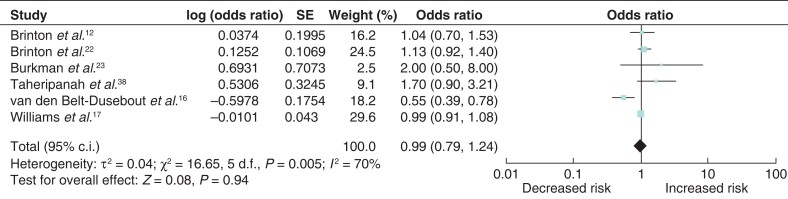
**Forest plot of breast-cancer risk associated with six or more** **cycles of *in vitro* fertilization**

To determine whether an increasing number of cycles of IVF were associated with excess breast-cancer risk, an analysis of six eligible studies was performed^12,16,17,22,23,38^. An increased incidence of breast cancer in women who received six or more cycles of fertility treatment was not observed (odds ratio 0.92 (95 per cent c.i. 0.73 to 1.16)). There was significant heterogeneity between the studies (*I*^2^ = 77%, *P* = 0.005) (*Fig. 7*).

### Long-term breast-cancer risk associated with fertility treatment

**Fig. 8:**
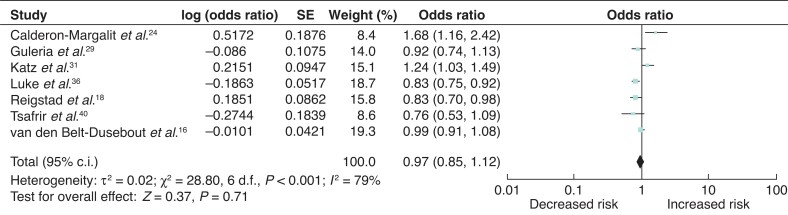
Forest plot of long-term breast-cancer risk associated with fertility treatment

Seven studies included patients with greater than 10 years’ follow-up^16,18,24,29,31,36,40^. There was no significant association between fertility treatment and excess breast-cancer risk in patients with greater than 10 years’ follow-up (OR 0.97 (95 per cent c.i. 0.85 to 1.12)) with significant heterogeneity between the studies (*I*^2^ = 79%, *P* = <0.001) (*Fig. 8*).

### Breast-cancer risk associated with IVF compared with subfertility reference group

**Fig. 9:**
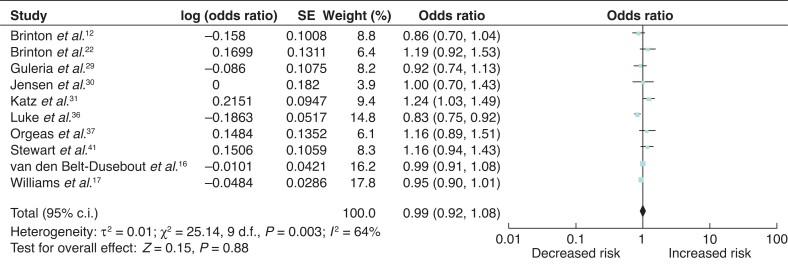
Forest plot of breast-cancer risk associated with *in vitro* fertilization using patients with subfertility as a reference group

Ten studies included women with subfertility as a reference group when analysing breast cancer risk^12,16,17,22,29–31,36,37,41^. As Jensen and colleagues did not report overall breast-cancer risk associated with all fertility treatment, the most commonly used regime described (clomiphene and gonadotropins) was included in this analysis^30^. There was no excess breast-cancer risk observed in this subgroup (odds ratio 0.99 (95 per cent c.i. 0.92 to 1.08); *I*^2^ = 64%, *P* = 0.003) (*Fig. 9*).

## Discussion

This is the largest meta-analysis in the past 20 years on the incidence of breast cancer associated with fertility treatment. There was no significant association demonstrated between fertility treatments and excess breast-cancer risk. The null hypothesis remained consistent when fertility treatment options such as clomiphene, gonadotropins, HcG and progesterone were examined individually.

IVF is a complex process involving several phases, treatment schedules and multiple drug regimens. Typically, in the first phase of controlled ovarian stimulation, a woman’s natural menstrual cycle is downregulated via administration of GnRH, followed by ovarian stimulation with gonadotropins causing a surge of oestrogen and progesterone levels^43^. If follicular development has occurred, HcG is administered 36 h prior to oocyte retrieval and progestogens or HcG are administered as luteal phase support for embryo implantation^16^. Clomiphene is the most commonly used agent to induce ovulation induction for women with anovulatory infertility and is often administered alone in the minimal ovarian-stimulation protocol for IVF^44,45^. Clomiphene is similar to tamoxifen as they are both selective oestrogen-receptor modulators. Selective oestrogen-receptor modulators are thought to act primarily by binding with oestrogen receptors in the hypothalamus and this competitive inhibition results in a perceived drop in endogenous oestrogen levels, eventually leading to increased gonadotrophin secretion and subsequent induction of ovulation^45^.

In contrast to the effects of tamoxifen, an elevated incidence of breast cancer treated with clomiphene was reported by Orgeas and colleagues in a cohort of 824 patients^40^. However, after adjusting for important confounding reproductive factors of age at first birth and total parity, the excess risk was only observed in women referred for infertility due to non-ovulatory factors who received four or more cycles of clomiphene (6 patients, standardised incidence ratio 1.90 (95 per cent c.i. 1.08 to 3.35))^40^. Findings from the present meta-analysis, however, did not find a significant association between clomiphene use and excess breast-cancer risk.

In 2007, Jensen and co-workers reported a four-fold increased risk of ductal breast cancer after use of progesterone^30^, however this was not reproducible in other studies^12,16,29^. Progesterone is mainly used as a routine treatment in most IVF/intracytoplasmic sperm injection protocols to enhance implantation of the fertilized eggs since it increases thickening of the endometrial lining^30^. As progesterone is often included as part of a regimen of multiple fertility drugs, the independent effect of progesterone on excess breast-cancer risk is difficult to establish. Jensen and co-workers also reported on the confounding potential of multiple administration of fertility drugs and concluded that the excess breast-cancer risk associated with progesterone use could be attributed to other types of fertility drugs and their combined effects^30^. The combined effects of progesterone with other fertility drugs did not appear to convey an increased risk of breast cancer in the present analysis.

HcG exerts its effect by triggering ovulation after induction of follicular growth and development with administration of gonadotropins. The role of HcG in breast cancer is paradoxical. Placental HcG acts as a protective agent of the mammary gland by determining a refractory condition to malignant transformation which is characterized by cellular differentiation, apoptosis and growth inhibition. Conversely, ectopic expression of β-HcG in various cancer entities is associated with a poor prognosis due to its tumour-promoting function^46^. Schüler-Toprak and colleagues suggest that mimicking pregnancy by treatment with HcG is a potential strategy for breast-cancer prevention^46^. There was no such protective association between breast-cancer risk and HcG noted in subgroup analyses of the included studies. Similarly, an adverse effect of gonadotropins on breast-cancer risk was reported by Burkman and co-workers^23^ and Taheripanah and colleagues^38^. Whilst gonadotropins do not directly affect breast tissue, they may increase circulating oestrogen levels during the follicular phase of ovulation-induction cycles and have an indirect influence on breast tissue^30^. Both Jensen and co-workers^30^ and Brinton and colleagues^47^ reported a higher breast-cancer risk associated with use of gonadotropin analogues among women who remain nulliparous but the lack of parity could be attributing to this excess risk.

One of the major challenges with studying the influence of fertility treatment on breast-cancer risk is the confounding associated with underlying causes of fertility. Nulliparity and infertility are associated with an increased risk of breast cancer, therefore comparisons with the general population may lead to inaccurate effect estimates^48^. Subgroup analysis of the 10 studies that used women with infertility problems as a reference group was performed to combat confounding from infertility and there was no excess risk observed. All studies used a retrospective study design to collect data and are therefore subject to information bias, as patients with infertility may have been exposed to alternative hormonal treatments outside of the remit of conventional IVF. The impact of such treatments on breast-cancer risk cannot be estimated due to study methodology limitations.

Breast-cancer incidence and associated death increase proportionally with age. Throughout the world, this disease peaks around age 60 years, with a sharp incline beginning at age 40^46^. Katz and colleagues studied a cohort of women who received a similar IVF regimen and compared breast-cancer incidence between those that did and did not develop breast cancer to identify women who are at increased risk of developing breast cancer after IVF^31^. Being aged over 30 at first IVF cycle was associated with a significantly elevated risk of developing breast cancer even after controlling for age at first pregnancy. Similarly Pappo and co-workers reported an increased incidence of breast cancer among women with hormonal infertility who were 40 or older and who underwent four or more IVF cycles^35^. They did not adjust for age at first pregnancy or address the fact that women over 40 having multiple cycles of IVF are likely to be nulliparous which could be contributing to their increased cancer risk. To determine whether there is a significant increased risk of cancer in women treated with IVF at an advanced age, Tsafrir and colleagues studied a cohort of 501 patients with a mean age at first IVF cycle of 42.3 years and greater than 10 years’ follow-up^40^. The incidence of breast cancer in women who received IVF was not associated with an excess risk at long-term follow-up. In contrast Stewart and colleagues compared breast-cancer incidence in a cohort of women undergoing treatment for infertility, comparing the rate in women who had IVF and those who did not and reported an increased rate of breast cancer in women who commenced IVF at a young age^41^. The authors highlighted the fact that information on other important risk factors for developing breast cancer, such as family history, germline mutation status and age of menarche, were not available and could have resulted in confounding of the results.

Burkmann and colleagues^23^ and Taheripanah and co-workers^38^ both reported an increased incidence of breast cancer with multiple IVF cycles (more than six cycles) and/or greater than 6 months of IVF treatment. However, it is worth noting that both studies collated data using non-specific patient questionnaires without medical record verification, which raises the possibility of recall bias. In the present meta-analysis, to elucidate whether there is a causative relationship between multiple IVF cycles and breast-cancer incidence, a subgroup analysis was performed which did not show a significant association between breast-cancer risk and six or more IVF cycles. Due to the growing evidence base in reproductive medicine, seven out of the 25 studies had greater than 10 years’ follow-up. Subgroup analyses of the studies with long-term follow-up did not demonstrate an association between excess breast-cancer risk and IVF use.

The potential risk of IVF treatment in the setting of germline mutations, such as breast cancer gene (*BRCA1* and *BRCA2*), was not analysed, however the evidence suggests that IVF is safe in patients with such mutations. A recent study performed by Derks-Smeets and colleagues on a cohort of 2514 women with *BRCA* mutations reported that no evidence was found for an association between ovarian stimulation for IVF and breast-cancer risk in *BRCA1/2*-mutation carriers^49^. Similarly, Kotsopoulos and colleagues performed a matched case–control study on 1380 pairs of women with *BRCA* mutations to determine if IVF was associated with an increased risk of breast cancer; no such association was found^50^. Provisional data from these studies suggest ovarian stimulation and IVF for fertility preservation due to *BRCA*-mutation status is safe. Similarly, studies which included patients with a previous history of breast cancer were not included in the present meta-analysis. Fertility preservation prior to commencement of chemotherapy is an option for women who wish to consider future pregnancies. Oocyte/embryo cryopreservation is the most well established and successful option for fertility preservation; it is, therefore, the recommended option for women with sufficient ovarian reserve who are medically stable to undergo controlled ovarian stimulation^51^. Ovarian stimulation causes an increase in the level of circulating oestrogen, and accordingly, many fertility-preservation programmes administer an aromatase inhibitor concurrently^51^. Due to the variability in regimes administered to women with a previous history of breast cancer, this cohort was omitted from the present meta-analysis.

There are several limitations to this study. Many of the studies included were retrospective cohort studies derived from national databases and therefore subject to recall and information bias. Data collated from the databases were often deficient of important information such as family history, HRT exposure, BMI, smoking, alcohol use and age of menarche, all of which are important risk factors for developing breast cancer and potential confounders. Twelve of the studies used the general population as a reference group. Women treated with IVF are likely to differ from the general population due to parity, age of first birth, age of menarche and age of menopause and these factors should be adjusted for when considering excess breast-cancer risk. Several large population-based studies with greater than 10 years’ follow-up were included in the analyses. One of the challenges with these studies is that during the study period, IVF regimens and strategies changed significantly as the number of ampoules of gonadotropins used increased until 1990 and decreased thereafter^16^. More recent IVF strategies largely consist of protocols with antagonists and shorter periods of downregulation, and improved success rates and therefore it is uncertain whether the results of this study can be generalizable to newer regimens.

This meta-analysis of 25 studies published in the past 20 years did not find a significant association between fertility treatments and excess breast-cancer risk. The same remained consistent when fertility treatment options such as clomiphene, gonadotropins, HcG and progesterone were examined individually. Women considering IVF should be informed that IVF does not appear to increase breast-cancer risk.

## Supplementary Material

zrab149_Supplementary_Data
