# Supplementary material for: Fertility treatment and breast-cancer incidence: meta-analysis
Source: BJS Open. 2022 Feb 9;6(1):zrab149. doi: 10.1093/bjsopen/zrab149 (PMC8830753; doi:10.1093/bjsopen/zrab149)
Supplement: zrab149_Supplementary_Data [file zrab149_supplementary_data.docx]

Table 1: Characteristics of Included Studies

| **Author** | **Country** | **Patients** | **Mean/**  **Median Age*** | **Study Period** | **Type of Fertility Treatment** | **ART implantation method** | **In Situ Lesions** | **Length of Follow up (year)** | **Reference Group** | **HR/**  **OR/**  **SIR** | **NOS** |
| --- | --- | --- | --- | --- | --- | --- | --- | --- | --- | --- | --- |
| Dor et al. 2001 | Israel | Fertility Rx: 5,026  Breast Ca: 11 | 34.0 +/- 6.4 | 1981-1996 | CC, Gtropins, GnRH agonist | Included | NR | 3.6 +/- 3.4 | General population | SIR | 6 |
| Doyle et al. 2002 | UK | Fertility Rx: 4,188  Breast Ca: 43 | 30.8 +/- 4.65 | 1975-1989 | CC, HcG, GnRH agonist | Not included | NR | NR | General population | SIR | 6 |
| Burkman et al. 2003 | US | Breast Ca: 4,575  Fertility Rx: 184 | NR | 1994-1998 | CC, Gtropins, HcG | NS | NR | NR | Women with breast Ca | OR | 7 |
| Lerner-Geva et al. 2003 | Israel | Fertility Rx: 1,082  Breast Ca: 5 | 32.7 +/- 4.8 | 1984-1996 | IVF- not specified | NS | NR | 6.5 | General population | SIR | 6 |
| Gauthier et al. 2004 | France | Fertility Rx: 6,602  Breast Ca: 183 | NR | 1990-2000 | CC, Gtropins, HcG | NS | All invasive | 9.7 | General population | RR | 6 |
| Terry et al. 2006 | US | Fertility Rx: NR  Breast Ca: 1,357 | NR | 1989-2001 | CC, Gtropins | NS | All invasive | NR | General population | HR | 7 |
| Kristiansson et al. 2006 | Sweden | Fertility Rx: 8,716  Breast Ca: 29 | NR | 1981-2001 | IVF-not specified | Included | 17% | 6.2 | General population | RR | 7 |
| Jensen et al. 2007 | Denmark | Fertility Rx: 54,362  Breast Ca: 331 | 30 | 1963-1998 | CC, Gtropins, GnRH, Progesterone | NS | All invasive | 8.8 | Infertile women | RR | 7 |
| Katz et al. 2008 | Israel | Fertility Rx: 7,162  Breast Ca: 41 | NR | 1984-2002 | IVF-not specified | NS | 10% | 12.9 | Infertile women | RR | 6 |
| Pappo et al. 2008 | Israel | Fertility Rx: 3,375  Breast Ca: 35 | 32.1 +/- 5.7 | 1986-2004 | IVF-not specified | NS | 14% | 8.1 +/- 4.3 | General population | SIR | 6 |
| Calderon-Margailt et al. 2008 | Israel | Fertility Rx: 14,463  Breast Ca: 498 | 27.5 | 1974-2004 | CC, Gtropins | NS | NR | 29 | General population | HR | 7 |
| Orgeas et al. 2009 | Sweden | Fertility Rx: 1,135  Breast Ca: 54 | 27 | 1961-2004 | CC, Gtropins | NS | NR | NR | Infertile women | SIR | 6 |
| Kallen et al. 2011 | Sweden | Fertility Rx: 24,058  Breast Ca: 95 | NR | 1982-2006 | IVF-not specified | NS | NR | 8.3 | General population | OR | 7 |
| Stewart et al. 2012 | Australia | Fertility Rx:7,381 Breast Ca: 148 | 31.3 +/- 5.1 | 1983-2010 | IVF-not specified | NS | 4.30% | 6.3 +/- 5.6 | Infertile women | HR | 7 |
| Yli-Kuha et al. 2012 | Finland | Fertility Rx: 9,175  Breast Ca: 55 | 33.5 | 1996-2004 | IVF- not specified | Included | NR | 7.8 | General population | OR | 8 |
| Brinton et al. 2013 | Israel | Fertility Rx: 67,608  Breast Ca: 389 | 31.1 | 1994-2011 | CC, Gtropins, Progesterone | NS | NR | 8.1 | Infertile women | HR | 7 |
| Brinton et al. 2014 | US | Fertility Rx: 9,872  Breast Ca: 749 | 30.1 | 1965-2010 | CC, Gtropins | NS | 22.9% | 30 | Infertile women | HR | 7 |
| Reigstad et al. 2015 | Norway | Fertility Rx: 16,626  Breast Ca: 138 | 32.5 | 1984-2010 | IVF- not specified | Included | NR | 16 | General population | HR | 7 |
| Van den Belt-Dussebout et al. 2016 | Netherlands | Fertility Rx: 19,158  Breast Ca: 948 | 32.8 | 1983-2013 | IVF-not specified | Included | 11.5% | 21.1 | Infertile women | HR | 8 |
| Luke et al. 2016 | USA | Fertility Rx: 6,690  Breast Ca: 112 | 35.3 +/- 5.3 | 2004-2009 | IVF- not specified | Included | NR | 11 | Infertile women | HR | 7 |
| Taheripanah et al. 2018 | Iran | Breast Ca: 928  Fertility Rx: 35 | 50.7 | 2011-2013 | CC, Gtropins, HcG | NS | 100% | NR | Women with breast Ca | OR | 7 |
| Fei et al. 2018 | US | Breast Ca: 1,422  Fertility Rx: 130 | 44.7 | 2008-2010 | Clomiphene, Gtropins | NS | 16% | NR | Women with breast Ca | OR | 6 |
| Williams et al. 2018 | UK | Fertility Rx: 255,786  Breast Ca: 2,576 | 34.5 | 1991-2010 | IVF- not specified | NS | NR | 8.8 | Infertile women | SIR | 8 |
| Guleria et al. 2019 | Denmark | Fertility Rx: 86,231  Breast Ca: 743 | 44.6 | 1995-2011 | CC,Gtropins  HcG,, Progesterone | NS | NR | 20.9 | General population (infertile subgroup analysis) | HR | 7 |
| Tsafrir et al. 2020 | Israel | Fertility Rx: 501  Breast Ca: 22 | 42.3 +/- 2.1 | 1994-2002 | IVF- not specified | NS | 23% | 16.7 +/- 3.7 | General population | SIR | 6 |

*Age of participants at start of study

Abbreviations: HR* Hazard ratio, RR* Risk ratio, OR* Odds ratio, SIR* Standardised incidence ratio, NOS* Nottingham Ottawa Score, Fertility Rx* Fertility treatment, ART* Assisted Reproductive Technology, NR* not reported, CC* Clomiphene citrate, IVF* In vitro fertilization, Gtropins* gonadotropins, GnRH* Gonadotropin releasing hormone, HcG* Human chorionic gonadotropin, NS* not specified
